
JOURNAL INFORMATION
==============================
NLM Title Abbreviation: ScientificWorldJournal
Journal ID: ScientificWorldJournal
NLM Journal ID: 101131163
Journal ID: TSWJ

The Scientific World Journal

ISSN: 2356-6140
EISSN: 1537-744X
Publisher: Wiley

ARTICLE INFORMATION
==============================
PMCID: PMC6083922
PMID: 30147553
DOI: 10.1100/tsw.2001.137
Article ID: 241832
Article version: 1
Subjects: Short Report

Gene expression profiling of Healthy Centenarians

Venanzoni M. 1 2
Franceschi C. 1 3
Sorbi S. 4
Storari L. 1
Giunta S. 1
Seth A. 5 *
1 Laboratory of Molecular Oncology, INRCA, Ancona, Italy
2 ITBM, CNR, Rome, Italy
3 University of Bologna, Italy
4 Dept. of Neurology and Psychiatrics, Univ. of Firenze, Italy
5 Dept. of Laboratory Medicine and Pathobiology, and Laboratory of Molecular Pathology, Sunnybrook and Women’s College Health Sciences Center, Univ. of Toronto, ON, Canada
*Corresponding author.
Publication date: 2001
Electronic publication date: 2001 Jul 17
Volume: 1
First page: 78
Last page: 78
Copyright: Copyright © 2001 M. Venanzoni et al.
Copyright year: 2001
License: This is an open access article distributed under the Creative Commons Attribution License, which permits unrestricted use, distribution, and reproduction in any medium, provided the original work is properly cited.
License URL: https://creativecommons.org/licenses/by/3.0/

==============================
Miami *Nature Biotechnology* Short Reports
*TheScientificWorld* (2001) 1(S3), 78SR
ISSN 1532-2246; DOI 10.1100/TSW.2001.137

### GENE EXPRESSION PROFILING OF HEALTHY CENTENARIANS

M. Venanzoni[1,2], C. Franceschi[1,3], S. Sorbi[4], L. Storari[1], S. Giunta[1] and A. Seth[5,]*

[1]Laboratory of Molecular Oncology, INRCA, Ancona, Italy; [2]ITBM, CNR, Rome, Italy; [3]Univ. of Bologna; [4]Dept. of Neurology and Psychiatrics, Univ. of Firenze, Italy; [5]Dept. of Laboratory Medicine and Pathobiology, and Laboratory of Molecular Pathology, Sunnybrook and Women's College Health Sciences Center, Univ. of Toronto, ON Canada
* Corresponding author.

**INTRODUCTION.** Human aging is characterized by a complex remodeling of immune, endocrine, metabolic parameters. In particular, organismal aging is characterized by high levels of proinflammatory molecules paralleling a progressive decay of response to stress at cellular and systemic level The few data on centenarian fibroblasts suggest that these cells possess only some features of replicative senescence, indicating that *in vitro* senescence plays a role, but do not fully mimic *in vivo* senescence. On the whole, aging is more appropriately to be considered as the consequence of the remodeling of an expression pattern involving a great number of genes. To compare the pattern of gene expression in centenarians *vs.* young control fibroblasts, an array of 384 genes was designed and cDNAs were robotically spotted. Genes were chosen on the basis of relationship with cell senescence, role in cellular stress response, involvement in apoptosis, genes involved in inflammation, tumor suppressor genes, cell cycle regulators, genes whose products have been demonstrated to change during aging, and genes whose genetic variants have been associated with human longevity.

**METHOD.** Primary cultures were established from skin fibroblasts from the forearms of 3 healthy female centenarians and 2 healthy female young controls (20 and 25 years old) from Central Italy. Total RNA was extracted by RNAzol B and further purified using Dynabeads coated with oligo-dT. Complex cDNA probes were produced by reverse transcription of mRNA followed by random primer labeling in presence of $^{32}$P-dCTP (1). Separate bacterial cultures harboring human cDNAs (I.M.A.G.E.) clones corresponding to known genes were robotically arrayed on nylon membranes (1). These were hybridized with the radiolabeled complex cDNA probes. The extent of hybridization at each spot was quantitated by phosphorimaging plate and by related software (1). Results are shown as intensity units for each cDNA relative to all other cDNAs.

**RESULTS AND DISCUSSION.** **Max** is one of the most up-regulated genes in fibroblasts from centenarians. Max forms heterodimers with Myc and regulates a variety of genes including telomerase, which affects the rate of aging in proliferative tissues. **Stress response pathways** were induced in centenarian fibroblasts as demonstrated by STAT, Heat Shock 70 and HSF expression. Surprisingly, p66SHC, the gene that affects life span in mice, is also expressed. **DNA damage inducible genes**, such as p53 and ATM, are induced, indicating that genotoxic stress occurs during aging. **TGFb**, TGFbR and activin receptors were induced, as was the IGF II receptor, an enhancer of TGF-beta response. **Cell cycle** related genes may be blocked, as $p57^{KIP2}$ was induced and Cyclins A, D1, F were down regulated, despite up-regulation of cdk4 and p35 cyclin H. **Apoptosis** may be promoted in centenarian fibroblasts given that Caspase3 was up-regulated, and the potent anti-apoptotic IL-15 was down-regulated (Table1).

*Table 1. Greatest change in relative intensity units of Centenarian vs. Youth*

| Increased in Centenarian Fibroblasts | Decreased in Centenarian Fibroblasts |
|---|---|
| p53 | Hic-5 |
| IGF-II receptor | Sm protein G |
| p35 cyclin H assembly factor | connective tissue GF |
| Max | PAK 1 |
| nerve growth factor receptor | BMP-1 |
| TRNR-2 | cyclin F |
| RNA helicase | TEK |
| Gos 3 [fosB] | abl interactor-2 (Abi-2) |
| alpha -1 type XVI collagen (COL16A1) | LnRNP-C |
| complement component 9 | cyclin A |
| complement component 8, beta polypeptide | Mcl-1 |
| Factor VII | type II serine/threonine kinase receptor |
| complement component 2 | IL-1 receptor type II |
| ATM | Sprk |
| ZO-1 | DLG-2 |
| Stat 5B | APO C III |
| Ski | jun D |
| heat shock factor 2 | IP gene |
| Id-1H | Thrombospondin |
| rap 1A | IL-10 receptor |

**REFERENCES.**

1. Seth, A., Lee, B.K., Qi, S., and Vary, C.P. (2000) J. Bone Miner. Res. 15(9), 1683-1696
